# An oleaginous yeast platform for renewable 1-butanol synthesis based on a heterologous CoA-dependent pathway and an endogenous pathway

**DOI:** 10.1186/s12934-018-1014-8

**Published:** 2018-10-25

**Authors:** Aiqun Yu, Yakun Zhao, Yaru Pang, Zhihui Hu, Cuiying Zhang, Dongguang Xiao, Matthew Wook Chang, Susanna Su Jan Leong

**Affiliations:** 1 State Key Laboratory of Food Nutrition and Safety, Key Laboratory of Industrial Fermentation Microbiology of the Ministry of Education, Tianjin Key Laboratory of Industrial Microbiology, College of Biotechnology, Tianjin University of Science and Technology, No. 29 the 13th Street TEDA, Tianjin, 300457 People’s Republic of China; 2 Department of Biochemistry, Yong Loo Lin School of Medicine, National University of Singapore, 8 Medical Drive, Singapore, 117597 Singapore; 3 NUS Synthetic Biology for Clinical and Technological Innovation (SynCTI), Life Sciences Institute, National University of Singapore, 28 Medical Drive, Singapore, 117456 Singapore; 4 Singapore Institute of Technology, 10 Dover Drive, Singapore, 138683 Singapore

**Keywords:** 1-butanol, Biosynthesis, CoA-dependent pathway, Metabolic engineering, *Y. lipolytica*

## Abstract

**Background:**

Microbial biofuel production provides a promising sustainable alternative to fossil fuels. 1-Butanol is recognized as an advanced biofuel and is gaining attention as an ideal green replacement for gasoline. In this proof-of-principle study, the oleaginous yeast *Yarrowia lipolytica* was first engineered with a heterologous CoA-dependent pathway and an endogenous pathway, respectively.

**Results:**

The co-overexpression of two heterologous genes *ETR1* and *EutE* resulted in the production of 1-butanol at a concentration of 65 μg/L. Through the overexpression of multiple 1-butanol pathway genes, the titer was increased to 92 μg/L. Cofactor engineering through endogenous overexpression of a glyceraldehyde-3-phosphate dehydrogenase and a malate dehydrogenase further led to titer improvements to 121 μg/L and 110 μg/L, respectively. In addition, the presence of an endogenous 1-butanol production pathway and a gene involved in the regulation of 1-butanol production was successfully identified in *Y. lipolytica*. The highest titer of 123.0 mg/L was obtained through this endogenous route by combining a pathway gene overexpression strategy.

**Conclusions:**

This study represents the first report on 1-butanol biosynthesis in *Y. lipolytica*. The results obtained in this work lay the foundation for future engineering of the pathways to optimize 1-butanol production in *Y. lipolytica*.

**Electronic supplementary material:**

The online version of this article (10.1186/s12934-018-1014-8) contains supplementary material, which is available to authorized users.

## Background

1-Butanol is an important advanced biofuel. Its energy content and characteristics are comparable with gasoline, which makes it an attractive alternative transportation fuel. This also makes 1-butanol a potential gasoline substitute, which is compatible with the existing infrastructure for fossil fuels [1]. The industrial production of 1-butanol by the fermentation of clostridial species is already established. Recently, several studies have demonstrated heterologous production of 1-butanol by introducing the clostridial CoA-dependent 1-butanol biosynthesis pathway into various microbial host strains [2–5]. In the CoA-dependent route, two molecules of acetyl-CoA are converted into a four-carbon chain butyryl-CoA for subsequent reduction to 1-butanol. Since acetyl-CoA is the precursor molecule for 1-butanol biosynthesis, the yield of 1-butanol is highly dependent upon the availability of acetyl-CoA. Therefore, it is beneficial to implement this pathway in production hosts with abundant intracellular acetyl-CoA, such as the oleaginous yeast *Yarrowia lipolytica*. Recently, an endogenous 1-butanol production pathway in yeast *Saccharomyces cerevisiae* was elucidated and the deletion of the alcohol dehydrogenase gene *ADH1* was found to efficiently activate 1-butanol production in *S. cerevisiae*. Thus, *ADH1* (or Adh1p) plays a central role in controlling the synthesis of 1-butanol through the endogenous pathway in *S. cerevisiae* [6]. The endogenous pathway was also proven to be more promosing than the CoA-dependent pathway for high-level production of 1-butanol in *S. cerevisiae*. Therefore, we also attempt to identify the presence of the endogenous 1-butanol production pathway similar to *S. cerevisiae* and the potential regulation pattern of 1-butanol synthesis in *Y. lipolytica*.

*Yarrowia lipolytica* naturally accumulates lipids at high amounts, i.e., up to 70% of cell dry weight and accumulates the lipids in its cytoplasm as triacylglycerols and stearoyl esters. These are subsequently metabolized to acetyl-CoA molecules via fatty acid oxidation in the peroxisome [7]. Thus, an ample supply of lipid-derived intracellular acetyl-CoA is available for the production of 1-butanol and other acetyl-CoA-derived bioproducts via the CoA-dependent pathway. This yeast strain can also be exploited to utilize a variety of low-cost and abundant feedstock, such as lipids from food waste, as carbon sources for growth and substrates for bioconversion [8, 9]. In addition to these unique features, a growing number of available genetic tools, protein expression systems and a fully sequenced genome make *Y. lipolytica* become one of the most widely studied unconventional yeast platform for microbial production of a wide variety of chemicals, via metabolic engineering strategies [10–15]. Herein, a proof-of-principle study on the metabolic engineering of *Y. lipolytica* for the production of 1-butanol via the heterologous CoA-dependent pathway was reported and the presence of an endogenous 1-butanol production pathway was also confirmed in *Y. lipolytica*.

## Results and discussion

### Construction of CoA-dependent 1-butanol pathway in *Y. lipolytica*

**Fig. 1:**
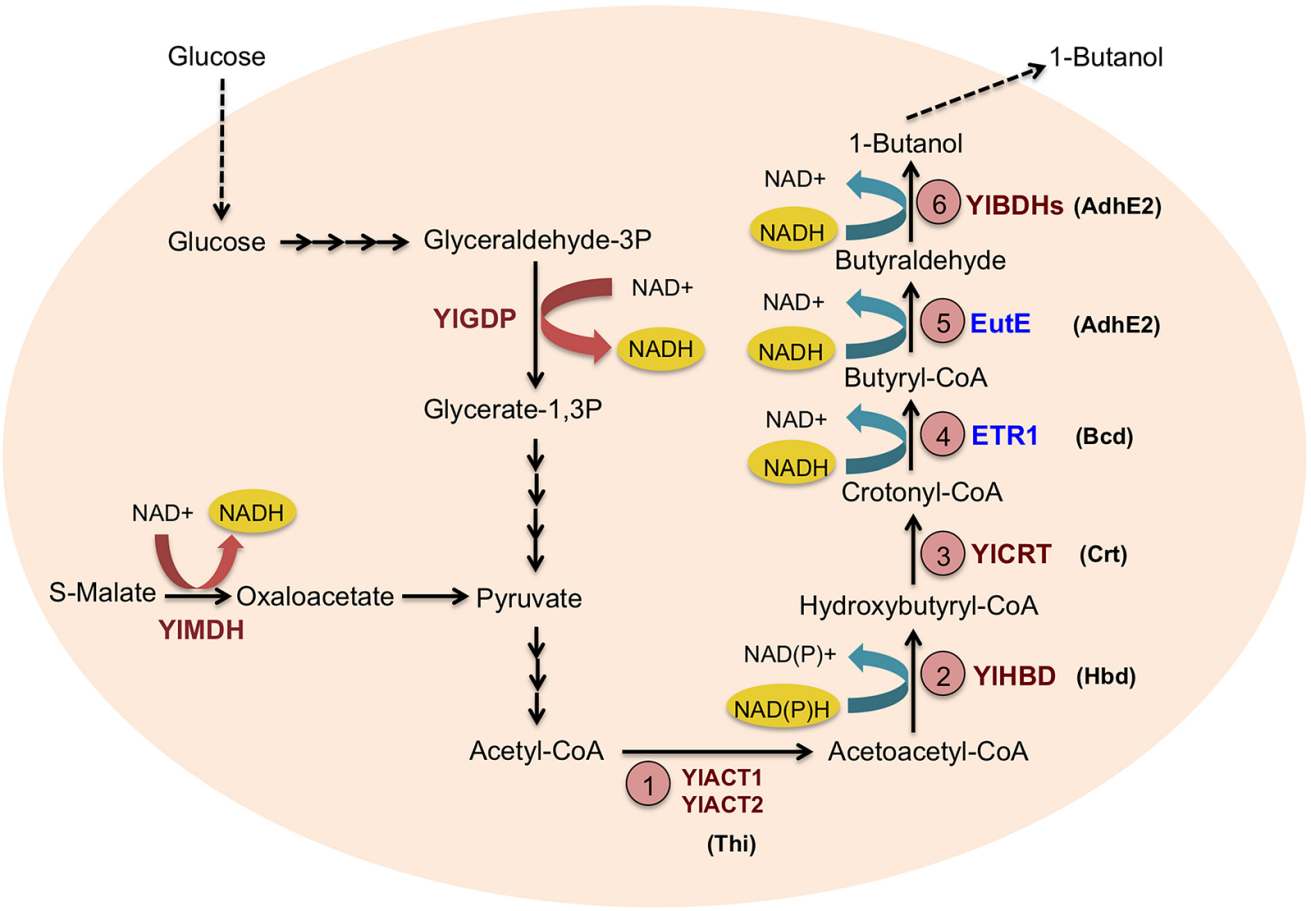
A schematic illustration of the reconstructed CoA-dependent 1-butanol pathway in engineered *Y. lipolytica* strains. Enzymes involved in the CoA-dependent 1-butanol pathway in *C. acetobutylicum* are shown in parentheses. Homologous enzymes found in *Y. lipolytica* are shown in red. To construct a complete 1-butanol pathway in *Y. lipolytica*, two heterologous genes, *ETR1* (from *S. cerevisiae*) and *EutE* (from *E. coli*), were introduced (shown in blue). The endogenous 1-butanol pathway enzymes that were overexpressed include *YlACT1*, *YlACT2*, *YlHBD* and *YlCRT* (red). *YlGPD* and *YlMDH* (red) were overexpressed for NADH regeneration in the engineered *Y. lipolytica* strains

The clostridial CoA-dependent 1-butanol biosynthesis pathway consists of six biochemical steps as illustrated in Fig. 1. To construct a pathway in *Y. lipolytica* for the conversion of acetyl-CoA to 1-butanol, a search of *Y. lipolytica* genome for a native CoA-dependent 1-butanol biosynthesis pathway homologous to that in *Clostridia* was first performed. Searching for alignment of the enzymes in the clostridial pathway was performed by using the Kyoto Encyclopedia of Genes and Genomes (KEGG) database and Basic Local Alignment Search Tool (BLAST). However, only a partial 1-butanol pathway containing four putative endogenous reactions homologous to steps 1, 2, 3 and 6 in the clostridial 1-butanol biosynthesis pathway was found in *Y. lipolytica*. The genes for the corresponding enzymes—*YlACT1*, *YlACT2*, *YLHBD*, *YlCRT* and several butanol dehydrogenase (i.e. *YlBDH*) candidates were also found in *Y. lipolytica*. *YlACT1* and *YlACT2* encode acetyl-CoA C-acyltransferases, which catalyze the initial step of the 1-butanol biosynthetic pathway for the conversion of two acetyl-CoA molecules to acetoacetyl-CoA. *YlHBD* encodes a 3-hydroxybutyryl-CoA dehydrogenase, which catalyzes the second step of the pathway, which converts reduced acetoacetyl-CoA to 3-hydroxybutyryl-CoA. The third step involves an enoyl-CoA hydratase encoded by *YlCRT* for the dehydration 3-hydroxybutyryl-CoA to crotonyl-CoA. For step 6, *YlBDH*s encode for butanol dehydrogenases that reduce butyraldehyde to the desired 1-butanol. Although the specific *YlBDH* has not been identified in this pathway, 1-butanol was detected when *Y. lipolytica* was fed with 0.1% butyraldehyde (data not shown). This indicates the presence of endogenous butanol dehydrogenase activity in *Y. lipolytica*, which can be attributed to the presence of numerous native alcohol dehydrogenases. In order to construct a complete 1-butanol biosynthesis pathway, enzymes capable of reducing crotonyl-CoA to butyryl-CoA (step 4 of the pathway) and its subsequent reduction to butyraldehyde (step 5 of the pathway) need to be introduced. This is because in the absence of heterologous enzymes to fulfill these roles, no 1-butanol was detected in the control strain Po1g::pYLEX1 (Additional file 1: Figure S1). To catalyze step 4, *ETR1* (the trans-2-enoyl-CoA reductase gene) from *S. cerevisiae* was chosen. Although *AdhE2* from *Clostridium acetobutylicum* encoding an aldehyde/alcohol dehydrogenase was commonly used for catalyzing step 5 of the pathway [2–5], this enzyme is oxygen-sensitive [16] and thus unsuitable for the pathway in *Y. lipolytica*, which is a strict aerobe. Therefore, the CoA-acylating aldehyde dehydrogenase gene, *EutE*, from *Escherichia coli* was selected, *as EutE* has been shown to be used successfully with *ETR1* to produce 1-butanol in *S. cerevisiae* [17]. *ETR1* and *EutE* were integrated into the chromosome of *Y. lipolytica* and overexpressed under the strong hybrid promoter hp4d. In this study, the resulting engineered strain, Po1g::pYLEX1-EuET, produced 1-butanol at a concentration of 65 μg/L, hence demonstrating the functional expression of *ETR1* and *EutE*. This also demonstrates the successful implementation of a CoA-dependent 1-butanol pathway, designed in this study, in *Y. lipolytica*. However, the titer obtained in the 1-butanol-producing Po1g::pYLEX1-EuET strain is low compared to other examples in the literatures on 1-butanol production in heterologous microbial hosts [2–5]. For example, by constructing the clostridial CoA-dependent pathway which contains the appropriate isozymes from a number of different organisms, 1-butanol was produced at a final titer of 2.5 mg/L in *S. cerevisiae* [5]. Therefore, this heterologous CoA-dependent 1-butanol biosynthesis pathway needs further optimization to boost product titer in *Y. lipolytica*.

### Gene overexpression to further improve 1-butanol production through the heterologous pathway

**Fig. 2:**
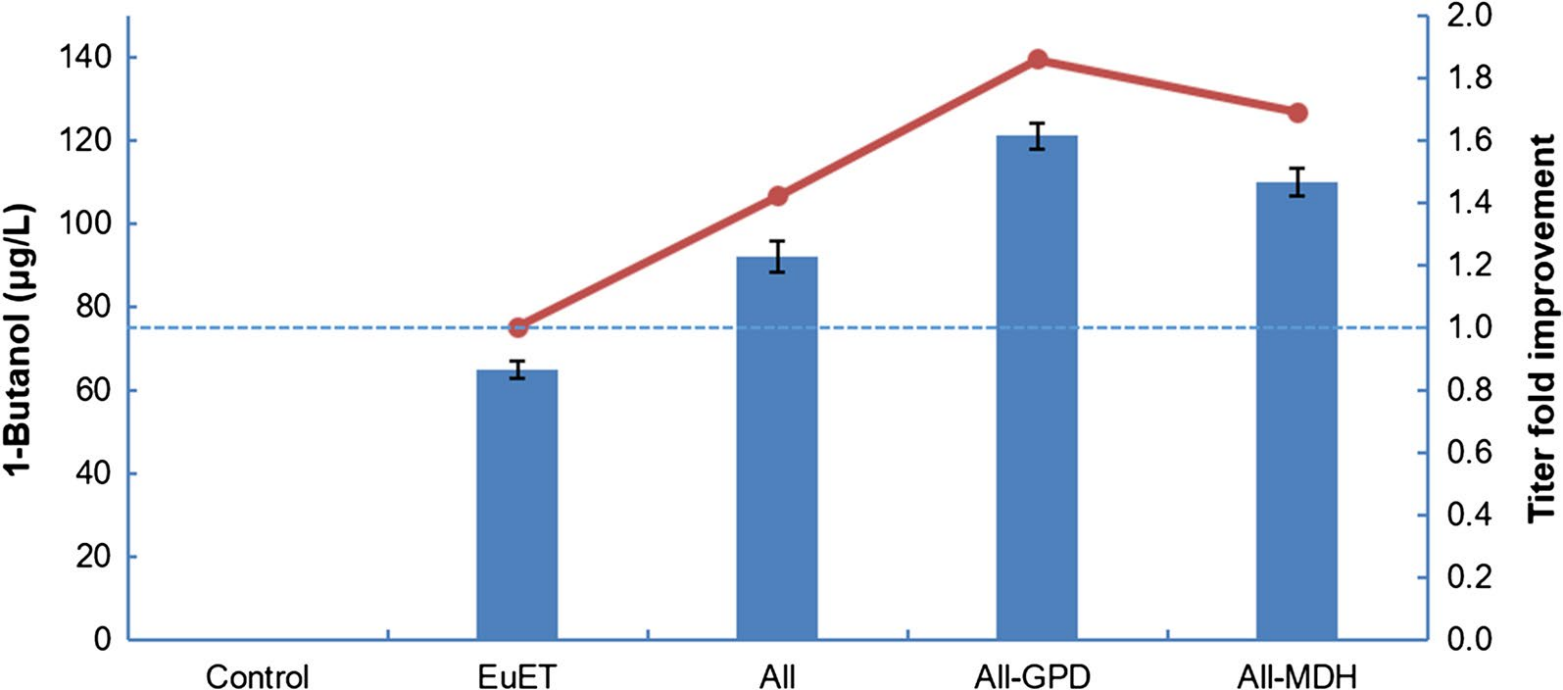
Production of 1-butanol in the engineered *Y. lipolytica* Po1g strains. All the engineered *Y. lipolytica* Po1g strains were cultivated aerobically at 30 °C, with vigorous shaking in YPD broth and 1-butanol titers were determined at the 24-h time point. Bars represent 1-butanol titers and lines represent 1-butanol titer improvements over EuET. Control, EuET, All, All-GPD and All-MDH refers to Po1g with pYLEX1 (empty vector), pYLEX1-EuET, pYLEX1-All, pYLEX1-All-GPD and pYLEX1-All-MDH, respectively. All values presented are the mean of three biological replicates ± standard deviation

To further enhance 1-butanol production following the successful construction of a functional 1-butanol biosynthetic pathway in *Y. lipolytica*, six heterologous and endogenous genes of the 1-butanol biosynthesis pathway were overexpressed. An additional copy each of *YlACT1*, *YlACT2*, *YlHBD* and *YlCRT* was integrated into the chromosome of *Y. lipolytica*, and overexpressed under the strong hybrid promoter hp4d along with *ETR1* and *EutE*. In doing so, a driving force for the synthesis of pathway intermediates will be created, and will thus direct the metabolic flux towards 1-butanol synthesis in *Y. lipolytica*. Consequently, the resulting Po1g::pYLEX1-All strain overexpressing the six abovementioned pathway genes achieved a titer of 92 μg/L 1-butanol, which is a 42% improvement compared to the Po1g::pYLEX1-EuET strain (Fig. 2).

We subsequently attempted to boost 1-butanol production by enhancing the recycling of reduced nicotinamide adenine dinucleotide (NADH), which is the cofactor that acts as the reducing agent in the 1-butanol biosynthesis pathway. In the constructed pathway designed in this study, four molecules of NADH are consumed for the production of one molecule of 1-butanol, along with concomitant formation of four molecules of oxidized nicotinamide adenine dinucleotide (NAD^+^). In several previous studies, increased NADH availability has been suggested to be important in achieving high titer production of 1-butanol via the CoA-dependent route [4, 18, 19]. Therefore, it is hypothesized that by overexpressing oxidative enzymes that utilize NAD^+^ as the cofactor, regeneration of NADH can be accelerated, thus providing sufficient supply of NADH for the 1-butanol biosynthesis pathway, which will thus enhance 1-butanol production. To this end, *YlGPD* and *YlMDH* were chosen for overexpression. *YlGPD* encodes for a glyceraldehyde-3-phosphate dehydrogenase that oxidizes glyceraldehyde-3-phosphate to 1,3-bisphosphoglycerate. *YlMDH* encodes for malate dehydrogenase that oxidizes (*S*)-malate to oxaloacetate. Both enzymes generate an NADH molecule from an NAD^+^ molecule per reaction, thus they can effectively recycle the NADH required for 1-butanol biosynthesis. Upon overexpression of *YlGPD* or *YlMDH*, in addition to the overexpression of six genes in Po1g::pYLEX1-All, 1-butanol titers of 121 μg/L and 110 μg/L were achieved after 24 h of cultivation which are 86% and 69% improvements compared to the Po1g::pYLEX1-EuET strain, respectively (Fig. 2). The enhanced titers demonstrated the efficacy of NADH regeneration by overexpression of *YlGPD* or *YlMDH* to boost 1-butanol production. Notably, longer cultivation time did not increase the 1-butanol titer.

However, there is still much room for improvement in the implementation of the heterologous CoA-dependent 1-butanol biosynthesis pathway in *Y. lipolytica*. A deeper understanding of the flux throughout this 1-butanol biosynthesis route is necessary to identify and eliminate the bottlenecks and competing pathways, in order to further improve 1-butanol accumulation in this oleaginous yeast system. For example, the enzymatic steps that limit the metabolic flux from acetyl-CoA to 1-butanol have to be determined in order to select the necessary genes for overexpression to direct the flux towards production of 1-butanol. Screening of alternative heterologous enzymes to substitute with *ETR1* and *EutE* may be worth pursuing, which will enhance catalysis of steps 4 and 5 in the pathway. To test a variety of isozyme candidates for each reaction in this pathway could also be one of the potential targets for greatly enhancing 1-butanol biosynthesis in engineered *Y. lipolytica*. Competing pathways also need to be identified so that the relevant genes can be deleted to accumulate 1-butanol and the intermediates. Additionally, other tools for protein overexpression in *Y. lipolytica*, such as multicopy integration of expression cassettes, can be employed to elevate the activities of the enzymes in the 1-butanol production pathway.

### Utilizing an endogenous pathway for 1-butanol production in *Y. lipolytica*

**Fig. 3:**
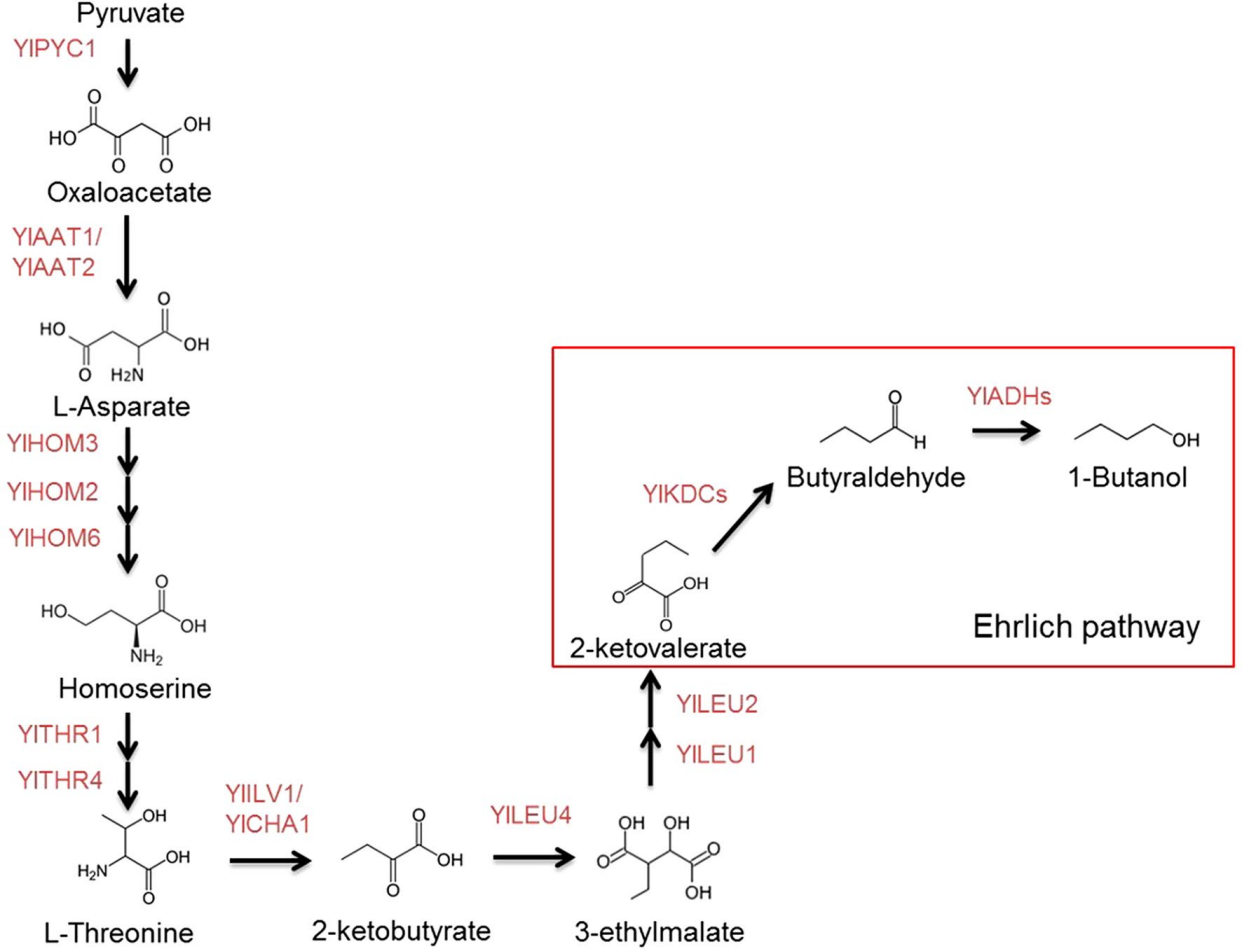
Proposed endogenous 1-butanol pathway in *Y. lipolytica*. This pathway and all the enzymes were predicted by homology search using Blastp program. All the *Y. lipolytica* enzymes are homologues to those in the endogenous 1-butanol production pathway of *S. cerevisiae*

Researchers recently reported the discovery of an endogenous 1-butanol production pathway in yeast *S. cerevisiae* and the alcohol dehydrogenase gene *ADH1* was proven to be able to regulate high-level production of 1-butanol in a manner that remains unclear in *S. cerevisiae* [6]. By homology search using Blastp program, we found all the *Y. lipolytica* enzymes that are homologues to those in the endogenous 1-butanol production pathway in *S. cerevisiae* (Fig. 3). Therefore, we attempted to create the alcohol dehydrogenase knockout mutant of *Y. lipolytica* to determine if the same mechanism is present within both yeast strains. Using the BLAST search tool, eleven putative alcohol dehydrogenase genes were identified in *Y. lipolytica* genome. The putative alcohol dehydrogenase genes are YALI0E17787g, YALI0D25630g, YALI0A16379g, YALI0E15818g, YALI0D02167g, YALI0A15147g, YALI0E07766g, YALI0F09603g, YALI0B10175g, YALI0F08129g and YALI0E07810g. These genes encode alcohol dehydrogenases which catalyze the reversible conversion between alcohols and aldehydes. We hypothesize that the deletion of these genes might lead to an accumulation of 1-butanol as happened with the deletion of *ADH1* in *S. cerevisiae*. As a result, we successfully deleted ten genes in parallel (except for YALI0A16379g) individually by using the disruption cassettes with long homologous sequences in the *Y. lipolytica* strain Po1g *KU70*Δ. Details of gene deletion experiments and results have already been described in our previous paper [20]. After confirming that the targeted gene had been deleted, the knockout mutant strains were then tested for 1-butanol production by using GC/MS. GC/MS analysis results indicated that 1-butanol was produced only in the YALI0E17787g-deletion strain at a titer of 100.2 mg/L (24 h) and with a productivity of 4.2 mg/L/h (Table 1). Moreover, GC/MS analysis results also indicated that 1-butanol was not detected in other *ADH* knockout strains (Table 1). The 1-butanol production in the YALI0E17787g-deletion strain also confirmed the presence of an endogenous 1-butanol production pathway in *Y. lipolytica*. To our knowledge, this is the first proposed endogenous metabolic pathway for 1-butanol production in *Y. lipolytica*. Furthermore, the involvement of the gene YALI0E17787g in activation of the endogenous 1-butanol biosynthetic pathway in *Y. lipolytica* was confirmed for the first time. It indicates this gene appears to perform a specific function in the process of 1-butanol production by *Y. lipolytica*. One possible reason why the deletion of YALI0E17787g can activate 1-butanol production is that the alcohol dehydrogenase encoded by YALI0E17787g is primarily responsible for 1-butanol oxidation in *Y. lipolytica*. Another reason is that metabolic flux is redirected towards 1-butanol production or the flux through the 1-butanol synthesis pathway is increased when YALI0E17787g was deleted. However, the mechanism of how YALI0E17787g acts as a critical regulator for turning on the endogenous 1-butanol pathway and regulating 1-butanol production in *Y. lipolytica* is not yet well understood because the underlying mechanism was not directly examined in this study. Obviously, further experiments are also required to fully investigate the underlying molecular mechanism of how YALI0E17787g functions in regulating 1-butanol production in *Y. lipolytica*. Moreover, exploring the mechanism underlying this phenomenon has great potential not only for biotechnological applications but also for advancing our scientific understanding of this unconventional yeast. The titer achieved from this endogenous pathway is much higher than that for the heterologous CoA-dependent pathway.

**Table 1:** The 1-butanol titers obtained in the *Y. lipolytica ADH* deletion strains

Strains	Titer (mg/L)	Productivity (mg/L/h)
*Y. lipolytica* Po1g	0	0
*Y. lipolytica* Po1g *KU70*Δ	0	0
*Y. lipolytica* Po1g *KU70*Δ YALI0E17787gΔ	100.2	4.2
*Y. lipolytica* Po1g *KU70*Δ YALI0D25630gΔ	0	0
*Y. lipolytica* Po1g *KU70*Δ YALI0E15818gΔ	0	0
*Y. lipolytica* Po1g *KU70*Δ YALI0D02167gΔ	0	0
*Y. lipolytica* Po1g *KU70*Δ YALI0A15147gΔ	0	0
*Y. lipolytica* Po1g *KU70*Δ YALI0E07766gΔ	0	0
*Y. lipolytica* Po1g *KU70*Δ YALI0F09603gΔ	0	0
*Y. lipolytica* Po1g *KU70*Δ YALI0B10175gΔ	0	0
*Y. lipolytica* Po1g *KU70*Δ YALI0F08129gΔ	0	0
*Y. lipolytica* Po1g *KU70*Δ YALI0E07810gΔ	0	0

The produced 1-butanol was quantified by GC/MS after 24 h of cultivation in shake flasks with YPD media. The *Y. lipolytica* Po1g strain and the *KU70* deletion platform strain were cultivated in parallel as control. All values presented are the mean of three biological replicates

Interestingly, it was found that 1-butanol accumulated mainly intracellularly when the heterologous CoA-dependent pathway was used. A possible reason for such a situation is that the resulting 1-butanol was trapped intracellularly due to the relatively low level of 1-butanol production. In contrast, 1-butanol accumulated mainly extracellularly upon application of the endogenous pathway. A possible reason for this is active secretion of 1-butanol into the culture medium when it was accumulated at a certain concentration inside the cells. Furthermore, the growth profiles of all the engineered strains were similar to that of the parental strain, indicating that the genetic modification of *Y. lipolytica* in this study did not cause any adverse effect on cell growth. The employment of the endogenous pathway could also avoid issues related to introduction of a heterologous pathway. Thus, this endogenous pathway is far more promosing than the CoA-dependent pathway for high-level production of 1-butanol in *Y. lipolytica*.

### Gene overexpression to further improve 1-butanol production through the endogenous pathway

We have shown that the deletion of the gene YALI0E17787g encoding alcohol dehydrogenase led to much higher levels of 1-butanol than the titer obtained by the CoA-dependent 1-butanol pathway. This result demonstrated that the engineering of the proposed endogenous pathway for 1-butanol production in *Y. lipolytica* is highly promising. We thus focused on the YALI0E17787g-deleted strain for our subsequent metabolic engineering efforts towards boosting 1-butanol production.

To further improve the production of 1-butanol in the YALI0E17787g-deleted strain, sixteen genes in the twelve steps of the proposed endogenous 1-butanol pathway (Fig. 3) were selected for overexpression. These genes encode different enzymes consisting of pyruvate carboxylase (PYC1, YALI0C24101g), aspartate aminotransferase (AAT1, YALI0B02178g and AAT1-2, YALI0F29337g), aspartate kinase (HOM3, YALI0D11704g), aspartate-semialdehyde dehydrogenase (HOM2, YALI0D13596g), homoserine dehydrogenase (HOM6, YALI0D01089g), homoserine kinase (THR1, YALI0F13453g), threonine synthase (THR4, YALI0F23221g), threonine dehydratase (ILV1, YALI0D02497g and CHA1, YALI0E10307g), 2-isopropylmalate synthase (LEU4, YALI0B07447g), 3-isopropylmalate dehydratase (LEU1, YALI0B01364g), 3-isopropylmalate dehydrogenase (LEU2, YALI0C00407g), alpha-keto-acid decarboxylases (ARO10, YALI0D06930g and PDC5, YALI0E07315g), pyruvate decarboxylase (PDC1, YALI0D10131g).

**Fig. 4:**
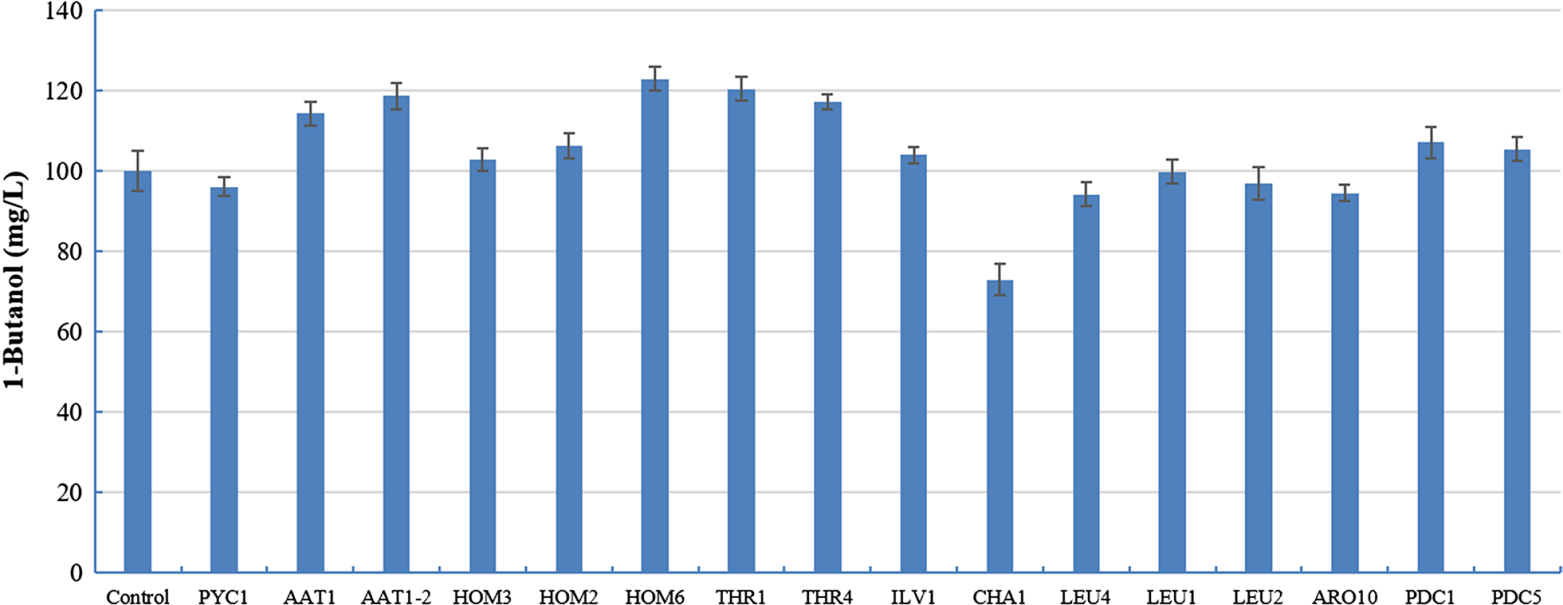
Effects of single-gene overexpression of genes in the endogenous 1-butanol pathway on 1-butanol production. Sixteen genes were overexpressed individually and titers of 1-butanol were quantified after 24 h of cultivation in shake flasks with YPD media. Glucose was used as the carbon source. The YALI0E17787g-deleted strain was cultivated in parallel as control. All values presented are the mean of three biological replicates ± standard deviation

All genes were overexpressed individually by integrating an additional copy of each gene into the chromosome of the YALI0E17787g-deleted strain. Individual overexpression of the selected genes did not lead to adverse effects on cell growth compared to the YALI0E17787g-deleted strain within 24 h of cultivation. The effects of overexpression of these 16 genes on overproduction of 1-butanol were investigated subsequently. The titers obtained in the gene-overexpression strains are shown in Fig. 4. It showed that the overexpression of five genes (*AAT1*, *AAT1*-*2*, *HOM6*, *THR1*, *THR4*) slightly improved the 1-butanol production compared to control. The *HOM6* overexpressed strain achieved a titer of 123.0 mg/L, which was a further 22.8% increase in 1-butanol titer over the YALI0E17787g-deleted strain. However, the overexpression of other genes only led to a marginal improvement, or even a little decrease. To further increase titer, the *HOM6* overexpressed YALI0E17787g-deleted strain with the highest titer 123.0 mg/L could be chosen for further engineering work.

## Conclusions

This proof-of-principle study describes the first biosynthesis of 1-butanol in the yeast *Y. lipolytica* via two independent routes: (1) the overexpression of two heterologous genes to complement a partially native 1-butanol CoA-dependent biosynthesis pathway and (2) engineering of an endogenous 1-butanol pathway. Furthermore, *Y. lipolytica* is an attractive host for the bioconversion of lipid-rich feedstock to value-added chemicals, owing to the yeast’s ability to utilize many hydrophobic substrates as carbon sources [8, 9]. Thus, the metabolically engineered *Y. lipolytica* strains described here provide a promising platform for the development of environmentally friendly 1-butanol production.

## Materials and methods

### Strains, media and culture conditions

*Escherichia coli* TOP10 and DH10B were used as the hosts for the cloning and propagation of plasmids. DH10B was especially used for cases with large plasmid constructs [21]. Both *E. coli* strains were routinely cultured in Luria–Bertani broth (LB) or on LB agar plates supplemented with 100 μg/mL of ampicillin at 37 °C. *Y. lipolytica* strain Po1g, a leucine auxotrophic derivative of the wild-type strain W29 (ATCC 20460), was used as the base strain in this study. *Y. lipolytica* strain Po1g *KU70*Δ was used as a platform strain when constructing other chromosomal deletion mutants. Routine cultivation of *Y. lipolytica* strains was carried out at 30 °C in yeast extract-peptone-dextrose (YPD) liquid media or on YPD agar plates. Synthetic complete media lacking leucine (YNBleu) were used for the selection of Leu^+^ transformants.

### DNA manipulation

**Fig. 5:**
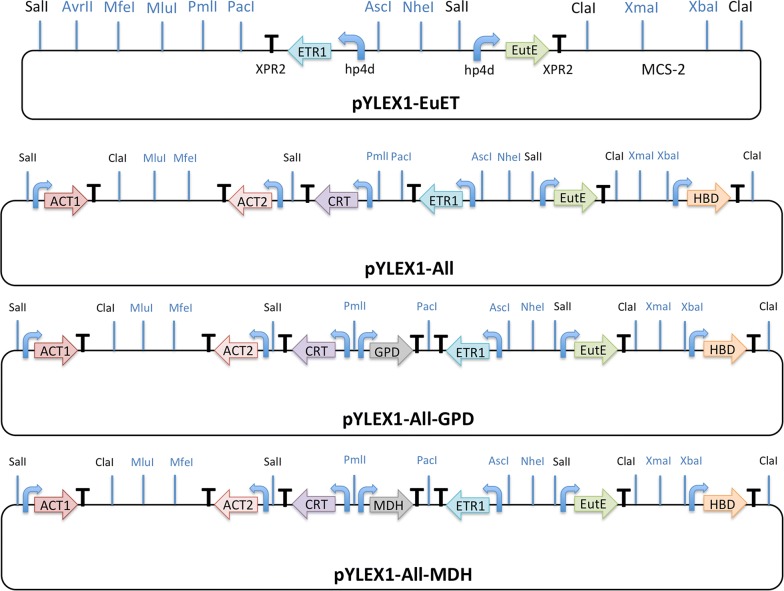
Plasmid maps of constructs containing gene integration cassettes used in this study. The vector pYLEX-EuET carries two heterologous genes *ETR1* and *EutE*. The vector pYLEX1-All was constructed through multiple rounds of restriction digestion and ligation for overexpressing the entire 1-butanol pathway. The vector pYLEX1-All-GPD carries all 1-butanol pathway genes and the *YlGPD* gene that encodes glyceraldehyde-3-phosphate dehydrogenase. The vector pYLEX1-All-MDH contains all 1-butanol pathway genes and the *YlMDH* gene that encodes malate dehydrogenase

Plasmid pYLEX1 (Yeastern Biotech, Taipei, Taiwan) containing the strong hybrid promoter (hp4d) was used for gene expression in *Y. lipolytica* Po1g. The plasmids constructed for the gene expression cassettes are graphically depicted in Fig. 5 and Additional file 1: Figures S2–S5. The primers used for gene cloning and plasmid construction are listed in Additional file 1: Table S1. In order to overexpress multiple genes in the *Y. lipolytica* Po1g strain, the original expression vector pYLEX1 was first modified by adding *Xma*I and *Xba*I sites into the *Cla*I site of pYLEX1, so as to generate a new multiple cloning site (MCS-2), yielding pYLEX1-2. Another pYLEX1 derivative, pYLEX1-3, was created by adding *Mlu*I, *Mfe*I and *Avr*II sites (MCS-3) to the *Cla*I site of pYLEX1 (Additional file 1: Figure S2). The *ETR1* gene from *S. cerevisiae* and *EutE* gene from *E. coli* were codon-optimized and synthesized by GenScript (Nanjing, China) for expression in *Y. lipolytica.* The native gene sets (i.e. *YlACT1*, *YlACT2*, *YlHBD*, *YlCRT*, *YlGPD* and *YlMDH*) were amplified from *Y. lipolytica* genomic DNA. The intron-containing genes, *YlACT1* and *YlCRT*, were assembled by overlap-extension PCR. The intron-containing gene *YlGPD* was amplified with a long forward primer. To clone the respective open reading frames of all these genes into pYLEX1, pYLEX1-2 or pYLEX1-3, an extra adenine nucleotide was added upstream of the start codons in the forward primers. Appropriate restriction sites were inserted downstream of the stop codons in the reverse primers. *ETR1* was ligated into the *Pml*I/*Bam*HI sites of pYLEX1 to yield pYLEX1-ETR1. *EutE* was ligated into the *Pml*I/*Bam*HI sites of pYLEX1-2 to yield pYLEX1-EutE. pYLEX1-EuET was constructed by PCR amplification of the *ETR1* gene cassette from pYLEX1-ETR1, using primers containing *Asc*I, *Nhe*I, *Avr*II, *Mfe*I, *Mlu*I, *Pml*I and *Pac*I sites, and then ligating into the *Sal*I site of pYLEX1-EutE (Fig. 5). *YlACT1* was ligated into the *Pml*I/*Bam*HI sites of pYLEX1 to yield pYLEX1-ACT1. *YlHBD* was ligated into the *Pml*I/*Bam*HI sites of pYLEX1 to yield pYLEX1-HBD. The gene cassette of *YlHBD* was amplified by PCR from pYLEX1-HBD, using primers containing *Mlu*I, *Mfe*I, *Xma*I and *Xba*I sites, and then ligated into the *Cla*I site of pYLEX1-ACT1 to yield pYLEX1-A1HB (Additional file 1: Figure S3). *YlACT2* was ligated into the *Pml*I/*Bam*HI sites of pYLEX1-3 to yield pYLEX1-ACT2. *YlCRT* was ligated into the *Pml*I/*Kpn*I sites of pYLEX1 to yield pYLEX1-CRT. The gene cassette of *YlCRT* was amplified by PCR from pYLEX1-CRT, using primers containing *Pac*I and *Pml*I sites, and then ligated into the *Sal*I site of pYLEX1-ACT2 to yield pYLEX1-A2CR (Additional file 1: Figure S4). Gene cassettes of *YlACT2* and *YlCRT* were digested from pYLEX1-A2CR with *Pac*I and *Mfe*I, and then ligated into the *Pac*I/*Mfe*I sites of pYLEX1-EuET to yield pYLEX1-A2CREuET (Additional file 1: Figure S5). Following the construction of pYLEX1-A2CREuET, gene cassettes of *YlACT2*, *YlCRT*, *ETR1* and *EutE* were digested from pYLEX1-A2CREuET using *Mfe*I and *Xma*I, and then ligated into the *Mfe*I/*Xma*I sites of pYLEX1-A1HB to yield pYLEX1-All (Fig. 5). The plasmid, pYLEX1-All, contains the gene cassettes of all six genes for building the clostridial 1-butanol pathway in *Y. lipolytica*. *YlGPD* was ligated into the *Pml*I/*Bam*HI sites of pYLEX1 to yield pYLEX1-GPD. *YlMDH* was ligated into the *Pml*I/*Bam*HI sites of pYLEX1 to yield pYLEX1-MDH. Gene cassettes of *YlGPD* and *YlMDH* were amplified by PCR from pYLEX1-GPD and pYLEX1-MDH, respectively, and then ligated into the *Pml*I/*Pac*I sites of pYLEX1-All. The resultant plasmids were designated as pYLEX1-All-GPD and pYLEX1-All-MDH, respectively (Fig. 5). A detailed procedure for the gene deletion in *Y. lipolytica* was already described in our previous paper [20]. In order to clone those endogenous genes into *Y. lipolytica* expression vector pYLEX1, another pYLEX1 derivative pYLEX1-NEW was created by adding *Xba*I, *Avr*II, *Mfe*I and *Mlu*I sites into the *Bam*HI site of pYLEX1 (Additional file 1: Figure S6). The subsequent construction process of pYLEX1-NEW recombinant vector series is the same as that of pYLEX1-EuET and pYLEX1-ETR1.

### Competent cell preparation and transformation of *Y. lipolytica* strains

Plasmids pYLEX1, pYLEX1-EuET, pYLEX1-A1A2CRHBEuET, pYLEX1-All-GPD, pYLEX1-All-MDH, were digested by *Spe*I, respectively. The linearized fragments were then integrated into the genome of the Po1g strain by transformation, using a protocol detailed in Additional file 1. The pYLEX1-NEW vector series were also first digested with Spe I and the resultant linearized fragments were integrated into the genome of the YALI0E17787g-deletion strain, in which the leucine selection marker was removed, by transformation using the same protocol as that employed to the Po1g strain.

### GC–MS analysis of 1-butanol produced in engineered *Y. lipolytica* strains

For the heterologous CoA-dependent pathway, the following engineered *Y. lipolytica* strains were generated: (a) Po1g::pYLEX1 (used as a negative control); (b) Po1g::pYLEX1-EuET; (c) Po1g::pYLEX1-All; (d) Po1g::pYLEX1-All-GPD; and (e) Po1g::pYLEX1-All-MDH. For the endogenous pathway, ten *Y. lipolytica* single-gene deletion mutants were generated [20]. Sixteen genes in the twelve steps of the proposed endogenous 1-butanol pathway were selected for overexpression and thus sixteen gene-overexpression strains were generated accordingly. To measure 1-butanol production, seed cultures were prepared by inoculating 5 mL of YPD medium in culture tubes with the engineered *Y. lipolytica* strains. The cells were grown overnight at 30 °C with agitation. Following that, 250-mL flasks containing 50 mL of YPD medium were inoculated to OD_600_ 0.05 with the seed cultures. All cultures were shaken at 225 rpm and 30 °C. Samples were then collected at 24 h, and 5 mL of each culture sample was centrifuged. Cell growth was monitored by OD_600_ measurement at every 2 h over 24 h. For the determination of extracellular 1-butanol, the product was extracted from the 2 mL supernatant by vortexing for 2 min with 2 mL of ethyl acetate. For the quantification of intracellular 1-butanol, the cell pellet from a 5-mL culture was lysed by eight rounds of 30-s bead beating with 1-min cooling on ice between each round. Following cell lysis, 1-butanol was extracted by vortexing for 2 min with 2 mL of ethyl acetate. The organic extracts were then analyzed by GC–MS using an HP 7890B GC with an Agilent 5977A MSD equipped with a DB-FFAP column (Agilent, Santa Clara, CA, USA). GC oven temperature was initially held at 45 °C for 2 min, and then ramped at 5 °C/min to 150 °C and held for 4 min. It was then subsequently ramped at 15 °C/min to 240 °C and held for 4 min. Helium was used as the carrier gas, with an inlet pressure of 13.8 psi. The injector was maintained at 250 °C and the ion source temperature was set to 230 °C. Final data analysis was achieved using Enhanced Data Analysis software (Agilent, Santa Clara, CA, USA). Isopropanol was used as an internal standard for the quantification of 1-butanol concentration.

## Additional file

**Additional file 1: Table S1.** Primers used for the construction of the heterologous CoA-dependent pathway. **Figure S1.** GC/MS detection of 1-butanol production in two *Y. lipolytica* engineered strains carrying the chromosomally integrated plasmid pYLEX1-All-GPD or pYLEX1-All-MDH. **Figure S2.** Map of the plasmids pYLEX1, pYLEX1-2 and pYLEX1-3. **Figure S3.** Map of the plasmid pYLEX1-A1HB. **Figure S4.** Map of the plasmid pYLEX1-A2CR. **Figure S5.** Map of the plasmid pYLEX1-A2CREuET. **Figure S6.** Restriction map of pYLEX1-NEW. Plasmid pYLEX1-NEW with an improved version of MCS was constructed by adding *Xba*I, *Avr*II, *Mfe*I and *Mlu*I sites into the *Bam*HI site of pYLEX1. Plasmid pYLEX1-NEW contains two *Bam*HI sites. Details on DNA sequence alignment and additional Materials and Methods are also provided as additional information.

## Abbreviations

GC/MS: gas chromatography mass spectrometry
OD_600_: optical density at 600 nm
KEGG: Kyoto Encyclopedia of Genes and Genomes
BLAST: Basic Local Alignment Search Tool
