# Supplementary material for: An oleaginous yeast platform for renewable 1-butanol synthesis based on a heterologous CoA-dependent pathway and an endogenous pathway
Source: Microb Cell Fact. 2018 Oct 25;17:166. doi: 10.1186/s12934-018-1014-8 (PMC6201493; doi:10.1186/s12934-018-1014-8)
Supplement: Supplementary file 1 — Additional file 1: Table S1. Primers used for the construction of the heterologous CoA-dependent pathway. Figure S1. GC/MS detection of 1-butanol production in two Y. lipolytica engineered strains carrying the chromosomally integrated plasmid pYLEX1-All-GPD or pYLEX1-All-MDH. Figure S2. Map of the plasmids pYLEX1, pYLEX1-2 and pYLEX1-3. Figure S3. Map of the plasmid pYLEX1-A1HB. Figure S4. Map of the plasmid pYLEX1-A2CR. Figure S5. Map of the plasmid pYLEX1-A2CREuET. Figure S6. Restriction map of pYLEX1-NEW. Plasmid pYLEX1-NEW with an improved version of MCS was constructed by adding XbaI, AvrII, MfeI and MluI sites into the BamHI site of pYLEX1. Plasmid pYLEX1-NEW contains two BamHI sites. Details on DNA sequence alignment and additional Materials and Methods are also provided as additional information. [file 12934_2018_1014_MOESM1_ESM.docx]

**Additional file**

**An oleaginous yeast platform for renewable 1-butanol synthesis based on a heterologous CoA-dependent pathway and an endogenous pathway**

Aiqun Yu, Yakun Zhao, Yaru Pang, Zhihui Hu, Cuiying Zhang, Dongguang Xiao, Matthew Wook Chang, Susanna Su Jan Leong

**Table S1.**

**Primers used for the construction of the heterologous CoA-dependent pathway**

| **Primers** | **Sequences(5’-3’)** | **Usage** |
| --- | --- | --- |
| 1 | CGATATATAT***CCCGGGTCTAGA***AT | Constructing the plasmid pYLEX1-2, top overlapping oligonucleotides. |
| 2 | CGAT***TCTAGACCCGGG***ATATATAT | Constructing the plasmid pYLEX1-2, bottom overlapping oligonucleotides. |
| 3 | CGAT***ACGCGTCAATTGCCTAGG***AT | Constructing the plasmid pYLEX1-3, top overlapping oligonucleotides. |
| 4 | CGAT***CCTAGGCAATTGACGCGT***AT | Constructing the plasmid pYLEX1-3, bottom overlapping oligonucleotides. |
| 5 | AATGGAGCCCGTCTACATTGT | Cloning the complete open reading frame (ORF) of *YlACT1* gene from *Y. lipolytica* Po1g genomic DNA, forward primer. |
| 6 | CGGGGGAGGGTACGTCTATATATAC | Cloning 5' end partial sequence of the first exon and the first exon of *YlACT1* gene from *Y. lipolytica* Po1g genomic DNA, forward primer. |
| 7 | TTGATGTCGGTCTTGGCGAGTGCGGCCTTCACGGCATGGGCTCCCAGATC | Cloning the first exon of *YlACT1* gene from *Y. lipolytica* Po1g genomic DNA, reverse primer. |
| 8 | GATCTGGGAGCCCATGCCGTGAAGGCCGCACTCGCCAAGACCGACATCAA | Cloning the second exon of *YlACT1* gene from *Y. lipolytica* Po1g genomic DNA, forward primer. |
| 9 | CG***GGATCC***CTAGTGGTGATGGTGATGATGACACTTCTCAACAATGATAG | Cloning the second exon and the complete ORF of *YlACT1* gene from *Y. lipolytica* Po1g genomic DNA, reverse primer. |
| 10 | AATGCGACTCACTCTGCCCCG | Cloning the ORF of *YlACT2* gene from *Y. lipolytica* Po1g genomic DNA, forward primer. |
| 11 | CG***GGATCC***CTAGTGGTGATGGTGATGATGCTCGACAGAAGAGACCTTCT | Cloning the ORF of *YlACT2* gene from *Y. lipolytica* Po1g genomic DNA, reverse primer. |
| 12 | AATGTTCCGACTCACCACTGC | Cloning the ORF of *YlHBD* gene from *Y. lipolytica* Po1g genomic DNA, forward primer. |
| 13 | CG***GGATCC***TTAGTGGTGATGGTGATGATG GTAGTCATAGAAGCCCTTTC | Cloning the ORF of *YlHBD* gene from *Y. lipolytica* Po1g genomic DNA, reverse primer. |
| 14 | AATGCGAAGCCTATATATAAACGTTCCGG | Cloning the complete ORF of *YlCRT* gene from *Y. lipolytica* Po1g genomic DNA, forward primer. |
| 15 | AAAGACAAATGACTAGCCGCCAGAC | Cloning 5' end partial sequence of the first exon and the first exon of *YlCRT* gene from *Y. lipolytica* Po1g genomic DNA, forward primer. |
| 16 | AGAGGAAGATCGGATGGTTCGGAGCATTTCTGTTCTGTGGTGGACTGTTTCTCG | Cloning the first exon of *YlCRT* gene from *Y. lipolytica* Po1g genomic DNA, reverse primer. |
| 17 | CGAGAAACAGTCCACCACAGAACAGAAATGCTCCGAACCATCCGATCTTCCTCT | Cloning the second exon of *YlCRT* gene from *Y. lipolytica* Po1g genomic DNA, forward primer. |
| 18 | GG***GGTACC***TTAGTGGTGATGGTGATGATGCTCGTTCTTGAAGTTGGGCT | Cloning the second exon and the complete ORF of *YlCRT* gene from *Y. lipolytica* Po1g genomic DNA, reverse primer. |
| 19 | AATGGCCATCAAAGTCGGTATTAACGGATTCGGACGAATCGGACGAATTGTCCTGCGAAACGCTCTCAAGAA | Cloning the ORF of *YlGPD* gene from *Y. lipolytica* Po1g genomic DNA, forward primer. |
| 20 | CG***GGATCC***CTAGTGGTGATGGTGATGATGAGCGGAAGCATCCTTCTTGGCGACGGCGAC | Cloning the ORF of *YlGPD* gene from *Y. lipolytica* Po1g genomic DNA, reverse primer. |
| 21 | AATGTTACGACTACGAACCATGCGACCCACA | Cloning the ORF of *YlMDH* gene from *Y. lipolytica* Po1g genomic DNA, forward primer. |
| 22 | CG***GGATCC***CTAGTGGTGATGGTGATGATGGTCGTAATCCCGCACATGGATGAGAGGCCG | Cloning the ORF of *YlMDH* gene from *Y. lipolytica* Po1g genomic DNA, reverse primer. |
| 23 | AATGCTGCCTACCTTCAAGCG | Cloning the ORF of *ScETR1* gene from artificial synthesized DNA fragments, forward primer. |
| 24 | CG***GGATCC***CTAGTGGTGATGGTGATGATGCCACTCCAGCACAACCATCT | Cloning the ORF of *ScETR1* gene from artificial synthesized DNA fragments, reverse primer. |
| 25 | AATGAACCAGCAGGATATTGA | Cloning the ORF of *EcEutE* gene from artificial synthesized DNA fragments, forward primer. |
| 26 | CG***GGATCC***CTAGTGGTGATGGTGATGATGCACAATTCGAAAAGCGTCCA | Cloning the ORF of *EcEutE* gene from artificial synthesized DNA fragments, reverse primer. |
| 27 | CGC***GTCGACGCTAGCGGCGCGCC***G CTCTCCCTTATGCGACTCC | Amplifying *ScETR1* expression cassette from pYLEX1-ETR1 into pYLEX1-EutE to yield pYLEX1-EuET, forward primer. |
| 28 | CGC***GTCGACCCTAGGCAATTGACGCGTCACGTGTTAATTAA***GAATTCGGACACGGGCATCT | Amplifying *ScETR1* expression cassette from pYLEX1-ETR1 into pYLEX1-EutE to yield pYLEX1-EuET, reverse primer. |
| 29 | CC***ATCGATACGCGTCAATTGCCCGGGTCTAGA***GCTCTCCCTTATGCGACTCC | Amplifying *YlHBD* expression cassette from pYLEX1-HBD into pYLEX1-ACT1 to yield pYLEX1-A1HB, forward primer. |
| 30 | CC***ATCGAT***GAATTCGGACACGGGCATCT | Amplifying *YlHBD* expression cassette from pYLEX1-HBD into pYLEX1-ACT1 to yield pYLEX1-A1HB, reverse primer. |
| 31 | CGC***GTCGACTTAATTAACACGTG*** GCTCTCCCTTATGCGACTCC | Amplifying *YlCRT* expression cassette from pYLEX1-CRT into pYLEX1-ACT2 to yield pYLEX1-A2CR, forward primer. |
| 32 | CGC***GTCGAC***GAATTCGGACACGGGCATCT | Amplifying *YlCRT* expression cassette from pYLEX1-CRT into pYLEX1-ACT2 to yield pYLEX1-A2CR, reverse primer. |
| 33 | ***GTG***GCTCTCCCTTATGCGACTCC | Amplifying *YlGPD* expression cassette from pYLEX1-GPD or *YlMDH* expression cassette from pYLEX1-MDH into pYLEX1-A1A2CRHBEuET to yield pYLEX1-All-GPD or pYLEX1-All-MDH, respectively, forward primer. |
| 34 | CC***TTAATTAA***GAATTCGGACACGGGCATCT | Amplifying *YlGPD* expression cassette from pYLEX1-GPD or *YlMDH* expression cassette from pYLEX1-MDH into pYLEX1-A1A2CRHBEuET to yield pYLEX1-All-GPD or pYLEX1-All-MDH, respectively, reverse primer. |

The restriction sites are in bold, italic and underlined.

**DNA alignment between codon-optimized and original sequence of *ETR1* gene**

Optimized 1 ATGCTGCCTACCTTCAAGCGATACATGTCTTCCTCCGCCCACCAGATTCCTAAGCATTTC

Original 1 ATGCTTCCCACATTCAAACGTTACATGTCGTCCTCAGCTCATCAGATTCCCAAGCACTTC

Optimized 61 AAGTCTCTCATTTACTCCACTCACGAGGTGGAGGACTGTACTAAGGTGCTGTCCGTCAAG

Original 61 AAATCGCTCATCTATTCAACTCATGAAGTTGAGGATTGTACCAAGGTTTTGTCAGTGAAA

Optimized 121 AACTACACCCCTAAGCAGGACCTCTCCCAGTCGATCGTTCTGAAGACCCTCGCCTTCCCC

Original 121 AATTATACGCCTAAACAAGACTTATCTCAATCAATTGTGTTAAAAACTTTGGCCTTTCCC

Optimized 181 ATCAACCCTTCTGACATTAACCAGCTCCAGGGAGTGTACCCCTCTCGACCTGAGAAGACT

Original 181 ATAAACCCTTCGGATATCAATCAGTTGCAAGGAGTATACCCGTCTCGTCCAGAAAAGACA

Optimized 241 TACGACTACTCCACCGATGAGCCCGCCGCTATTGCCGGCAACGAGGGAGTGTTCGAGGTG

Original 241 TACGATTACTCCACAGATGAGCCAGCCGCTATCGCCGGTAATGAGGGTGTCTTTGAAGTT

Optimized 301 GTCTCGCTGCCTTCTGGATCTTCCAAGGGTGACCTGAAGCTCGGAGATCGAGTCATCCCC

Original 301 GTTTCTTTACCTTCGGGAAGTTCCAAGGGAGATTTGAAATTGGGTGACCGAGTTATCCCA

Optimized 361 CTCCAGGCTAACCAGGGCACCTGGTCCAACTACCGAGTGTTCTCTTCTTCTTCTGACCTG

Original 361 TTGCAGGCAAATCAAGGGACTTGGTCCAATTATAGAGTTTTCTCTAGTAGTTCTGATTTA

Optimized 421 ATTAAGGTGAACGACCTGGATCTCTTCTCCGCCGCTACTGTCTCGGTTAACGGTTGCACC

Original 421 ATCAAGGTAAATGATTTGGATCTGTTTTCTGCGGCAACTGTATCTGTTAATGGTTGTACC

Optimized 481 GGCTTTCAGCTCGTCTCGGACTACATCGATTGGAACTCTAACGGTAACGAGTGGATCATT

Original 481 GGTTTCCAATTAGTATCAGACTATATCGACTGGAACAGTAACGGTAATGAATGGATTATC

Optimized 541 CAGAACGCCGGCACTTCTTCCGTGTCGAAGATTGTTACCCAGGTGGCCAAGGCTAAGGGC

Original 541 CAAAATGCCGGTACATCTAGTGTATCAAAAATAGTTACGCAAGTAGCAAAAGCTAAAGGG

Optimized 601 ATCAAGACCCTGTCTGTCATTCGAGACCGAGATAACTTCGACGAGGTCGCCAAGGTGCTG

Original 601 ATCAAAACATTAAGTGTTATACGTGACCGTGATAATTTTGATGAGGTAGCAAAAGTTTTG

Optimized 661 GAGGATAAGTACGGAGCTACTAAGGTCATCTCTGAGTCCCAGAACAACGACAAGACCTTT

Original 661 GAGGATAAGTATGGTGCTACGAAGGTTATTTCCGAATCGCAAAACAACGACAAGACTTTT

Optimized 721 GCCAAGGAGGTTCTGTCCAAGATTCTCGGAGAGAACGCCCGAGTGCGACTGGCTCTCAAC

Original 721 GCCAAAGAAGTATTGTCCAAGATTTTGGGTGAAAATGCAAGGGTGAGGCTTGCCTTGAAT

Optimized 781 TCCGTCGGCGGAAAGTCGTCTGCCTCGATCGCTCGAAAGCTGGAGAACAACGCTCTGATG

Original 781 TCTGTTGGAGGTAAATCCAGTGCATCAATAGCACGTAAGTTGGAAAATAATGCTTTGATG

Optimized 841 CTCACTTACGGTGGCATGTCGAAGCAGCCCGTCACTCTGCCTACCTCTCTCCACATTTTC

Original 841 CTCACTTATGGAGGAATGTCAAAACAACCTGTAACTTTACCAACATCTCTACACATTTTC

Optimized 901 AAGGGCCTGACTTCCAAGGGATACTGGGTTACCGAGAAGAACAAGAAGAACCCTCAGTCT

Original 901 AAAGGCTTGACATCCAAAGGGTACTGGGTGACTGAAAAGAACAAAAAAAACCCCCAAAGC

Optimized 961 AAGATCGACACCATTTCCGATTTTATCAAGATGTACAACTACGGACATATCATTTCTCCC

Original 961 AAGATTGACACCATCAGTGATTTTATCAAAATGTATAATTATGGTCACATTATTTCACCA

Optimized 1021 CGAGACGAGATTGAGACTCTGACCTGGAACACTAACACCACTACCGATGAGCAGCTGCTG

Original 1021 AGAGATGAAATTGAAACTCTTACCTGGAATACTAACACTACTACTGACGAACAGTTACTA

Optimized 1081 GAGCTGGTGAAGAAGGGCATCACCGGAAAGGGCAAGAAGAAGATGGTTGTGCTGGAGTGG

Original 1081 GAACTAGTCAAAAAAGGTATAACTGGGAAGGGGAAGAAAAAAATGGTTGTTTTAGAATGG

Optimized 1141 TAG

Original 1141 TAA

**DNA alignment between codon-optimized and original sequence of *EutE* gene**

Optimized 1 ATGAACCAGCAGGATATTGAGCAGGTCGTGAAGGCTGTTCTCCTGAAGATGCAGTCGTCC

Original 1 ATGAATCAACAGGATATTGAACAGGTGGTGAAAGCGGTACTGCTGAAAATGCAAAGCAGT

Optimized 61 GATACCCCCTCCGCCGCCGTCCATGAGATGGGAGTGTTCGCCTCGCTGGACGATGCTGTC

Original 61 GACACGCCGTCCGCCGCCGTTCATGAGATGGGCGTTTTCGCGTCCCTGGATGACGCCGTT

Optimized 121 GCCGCTGCCAAGGTTGCCCAGCAGGGTCTGAAGTCTGTCGCCATGCGACAGCTCGCTATC

Original 121 GCGGCAGCCAAAGTCGCCCAGCAAGGGTTAAAAAGCGTGGCAATGCGCCAGTTAGCCATT

Optimized 181 GCTGCCATTCGAGAGGCTGGAGAGAAGCATGCCCGAGACCTGGCTGAGCTGGCCGTCTCC

Original 181 GCTGCCATTCGTGAAGCAGGCGAAAAACACGCCAGAGATTTAGCGGAACTTGCCGTCAGT

Optimized 241 GAGACCGGTATGGGTCGAGTTGAGGATAAGTTTGCCAAGAACGTGGCTCAGGCCCGAGGC

Original 241 GAAACCGGCATGGGGCGCGTTGAAGATAAATTTGCAAAAAACGTCGCTCAGGCGCGCGGC

Optimized 301 ACCCCCGGCGTTGAGTGTCTGTCGCCTCAGGTGCTCACCGGAGACAACGGTCTGACTCTC

Original 301 ACACCAGGCGTTGAGTGCCTCTCTCCGCAAGTGCTGACTGGCGACAACGGCCTGACCCTA

Optimized 361 ATCGAGAACGCCCCCTGGGGAGTGGTCGCTTCTGTGACCCCCTCCACTAACCCTGCTGCC

Original 361 ATTGAAAACGCACCCTGGGGCGTGGTGGCTTCGGTGACGCCTTCCACTAACCCGGCGGCA

Optimized 421 ACCGTCATCAACAACGCCATCTCCCTGATTGCTGCCGGTAACTCGGTTATTTTCGCTCCC

Original 421 ACCGTAATTAACAACGCCATCAGCCTGATTGCCGCGGGCAACAGCGTCATTTTTGCCCCG

Optimized 481 CACCCTGCTGCCAAGAAGGTCTCGCAGCGAGCCATCACCCTGCTCAACCAGGCTATTGTG

Original 481 CATCCGGCGGCGAAAAAAGTCTCCCAGCGGGCGATTACGCTGCTCAACCAGGCGATTGTT

Optimized 541 GCTGCCGGCGGACCTGAGAACCTGCTCGTCACCGTTGCCAACCCTGACATCGAGACTGCT

Original 541 GCCGCAGGTGGGCCGGAAAACTTACTGGTTACTGTGGCAAATCCGGATATCGAAACCGCG

Optimized 601 CAGCGACTGTTCAAGTTTCCTGGAATTGGTCTGCTCGTTGTGACCGGTGGAGAGGCTGTC

Original 601 CAACGCTTGTTCAAGTTTCCGGGTATCGGCCTGCTGGTGGTAACCGGCGGCGAAGCGGTA

Optimized 661 GTTGAGGCTGCTCGAAAGCATACTAACAAGCGACTGATCGCTGCCGGCGCTGGAAACCCT

Original 661 GTAGAAGCGGCGCGTAAACACACCAATAAACGTCTGATTGCCGCAGGCGCTGGCAACCCG

Optimized 721 CCTGTGGTCGTTGACGAGACCGCCGATCTGGCCCGAGCCGCTCAGTCGATCGTGAAGGGA

Original 721 CCGGTAGTGGTGGATGAAACCGCCGACCTCGCCCGTGCCGCTCAGTCCATCGTCAAAGGC

Optimized 781 GCCTCTTTTGACAACAACATCATTTGCGCTGATGAGAAGGTCCTGATTGTGGTGGACTCT

Original 781 GCTTCTTTCGATAACAACATCATTTGTGCCGACGAAAAGGTACTGATTGTTGTTGATAGC

Optimized 841 GTTGCCGATGAGCTGATGCGACTCATGGAGGGCCAGCACGCTGTGAAGCTGACCGCCGAG

Original 841 GTAGCCGATGAACTGATGCGTCTGATGGAAGGCCAGCACGCGGTGAAACTGACCGCAGAA

Optimized 901 CAGGCTCAGCAGCTCCAGCCTGTCCTGCTCAAGAACATTGACGAGCGAGGAAAGGGCACC

Original 901 CAGGCGCAGCAGCTGCAACCGGTGTTGCTGAAAAATATCGACGAGCGCGGAAAAGGCACC

Optimized 961 GTGTCCCGAGACTGGGTCGGTCGAGATGCTGGAAAGATCGCTGCCGCTATTGGACTGAAG

Original 961 GTCAGCCGTGACTGGGTTGGTCGCGACGCAGGCAAAATCGCGGCGGCAATCGGCCTTAAA

Optimized 1021 GTCCCCCAGGAGACCCGACTGCTCTTCGTTGAGACCACTGCCGAGCACCCTTTTGCTGTG

Original 1021 GTTCCGCAAGAAACGCGCCTGCTGTTTGTGGAAACCACCGCAGAACATCCGTTTGCCGTG

Optimized 1081 ACTGAGCTGATGATGCCCGTCCTCCCTGTTGTGCGAGTTGCCAACGTGGCCGACGCTATC

Original 1081 ACTGAACTGATGATGCCGGTGTTGCCCGTCGTGCGCGTCGCCAACGTGGCGGATGCCATT

Optimized 1141 GCCCTGGCTGTGAAGCTGGAGGGAGGTTGTCACCATACCGCCGCTATGCATTCTCGAAAC

Original 1141 GCGCTAGCGGTGAAACTGGAAGGCGGTTGCCACCACACGGCGGCAATGCACTCGCGCAAC

Optimized 1201 ATTGAGAACATGAACCAGATGGCCAACGCTATCGATACTTCCATTTTCGTCAAGAACGGT

Original 1201 ATCGAAAACATGAACCAGATGGCGAATGCTATTGATACCAGCATTTTCGTTAAGAACGGA

Optimized 1261 CCTTGCATCGCTGGACTGGGTCTCGGTGGAGAGGGTTGGACCACTATGACCATTACCACT

Original 1261 CCGTGCATTGCCGGGCTGGGGCTGGGCGGGGAAGGCTGGACCACCATGACCATCACCACG

Optimized 1321 CCTACTGGCGAGGGAGTTACCTCCGCCCGAACTTTCGTGCGACTGCGACGATGTGTGCTC

Original 1321 CCAACCGGTGAAGGGGTAACCAGCGCGCGTACGTTTGTCCGTCTGCGTCGCTGTGTATTA

Optimized 1381 GTGGACGCTTTTCGAATTGTGTAG

Original 1381 GTCGATGCGTTTCGCATTGTTTAA

**Additional Materials and Methods**

**Reagents**

iProof high-fidelity DNA polymerase was purchased from Bio-Rad Labs (Hercules, CA, USA). Restriction enzymes, T4 DNA ligase, Taq DNA polymerase and PCR reagents were purchased from New England Biolabs (Beverly, MA, USA). CSM-Leu (complete supplement mixture minus leucine) drop-out mixture was purchased from MP Biomedicals (Solon, OH, USA). Yeast extract and peptone were purchased from BD Biosciences (San Jose, CA, USA). Oligonucleotides were synthesized by Integrated DNA Technologies (Singapore). All other reagents were purchased from Sigma Aldrich (St. Louis, MO, USA) unless otherwise stated.

**Preparation of *Y. lipolytica* Po1g competent cell**

1. Inoculate a colony of *Y. lipolytica* Po1g strain from a fresh YPD plate in 10 mL YPD medium (1% yeast extract, 2% peptone, 2% dextrose and 50 mM citrate buffer pH 4.0) in a 250 mL flask. Incubate with shaking at 225 rpm at 30°C for 20 hours.

2. Pellet the cells by centrifuging 5 minutes at 5,000 g at room temperature.

3. Wash the cells with 20 mL TE buffer and pellet the cells similarly as Step 2.

4. Resuspend the cells in 1 mL of 0.1 M lithium acetate (pH 6.0, adjusted with acetic acid) and incubate for 10 minutes at room temperature.

5. Aliquot the competent cells (100 µL) into sterile 1.5 mL tubes. Proceed to the transformation steps below immediately, or add glycerol to a final concentration of 25% (v/v) and store at -80°C for long-term storage.

**Transformation of *Y. lipolytica* Po1g cells**

1. Gently mix 10 µL of denatured salmon sperm DNA (10 mg/mL) and 1-5 µg of the linearized plasmid together with 100 µL of competent cells, and incubate at 30°C for 15 minutes.

2. Add 700 µL of 40% PEG-4000 (dissolved in 0.1M lithium acetate pH 6.0), mix well and incubate at 30°C for 60 minutes with shaking (225 rpm).

3. Heat shock the transformation mixture at 39°C for 60 minutes.

4. Add 1 mL YPD medium and recover for 2 hours at 30°C and 225 rpm.

5. Centrifuge at 10,000 g for 1 minute, remove supernatant and resuspend the pellet in 1 mL of TE buffer.

6. Repellet the cells and discard supernatant again.

7. Resuspend the pellet in 100 µL of TE buffer and plate onto selective plates (leucine-deficient plates).

**Additional Figures**


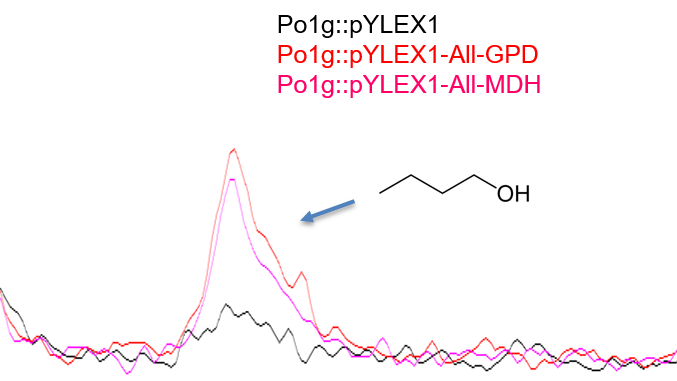


**Figure S1.** GC/MS detection of 1-butanol production in two *Y. lipolytica* engineered strains carrying the chromosomally integrated plasmid pYLEX1-All-GPD or pYLEX1-All-MDH.


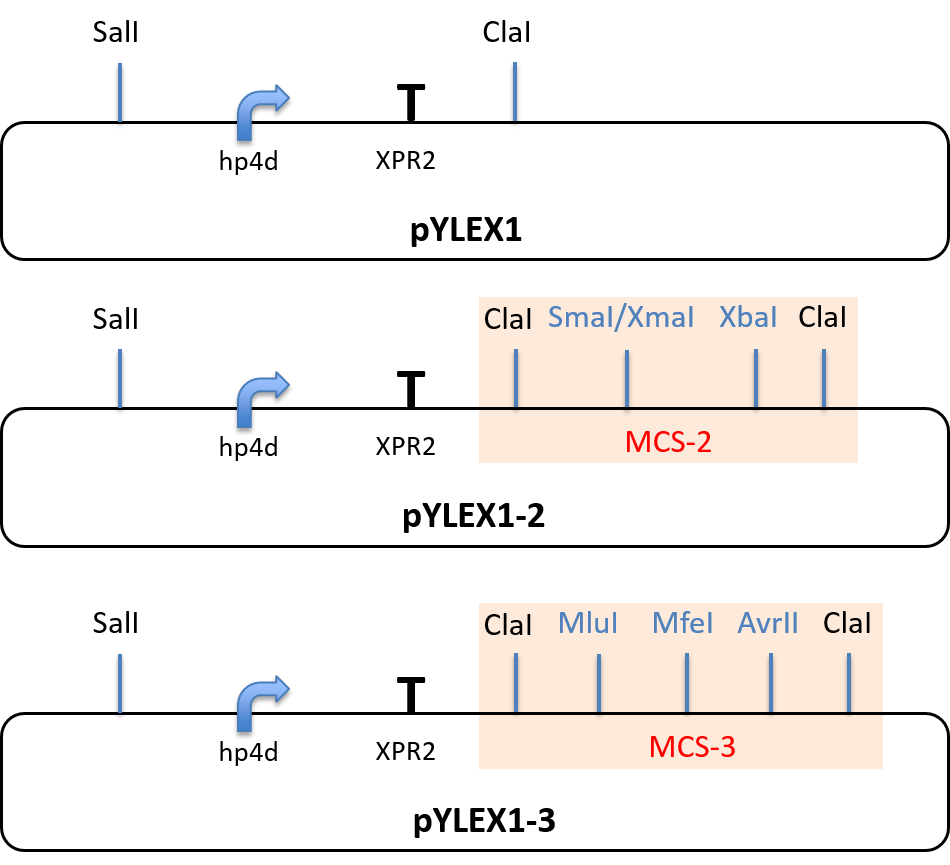


**Figure S2.** Map of the plasmids pYLEX1, pYLEX1-2 and

pYLEX1-3


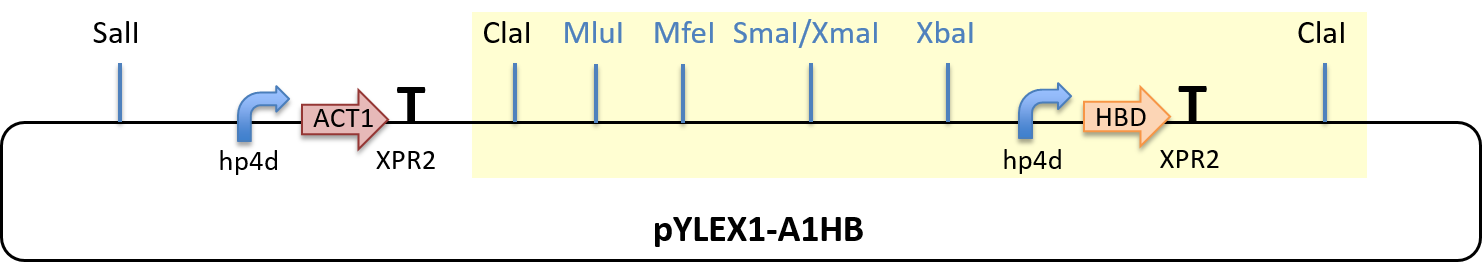


**Figure S3.** Map of the plasmid pYLEX1-A1HB


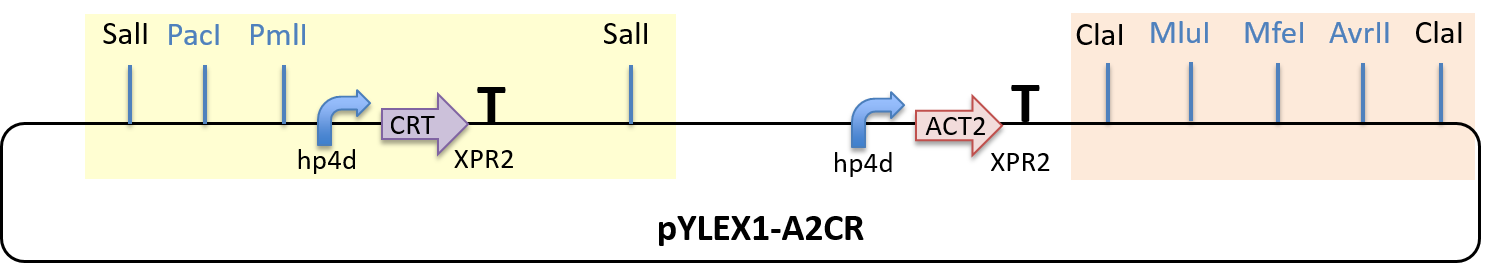


**Figure S4.** Map of the plasmid pYLEX1-A2CR


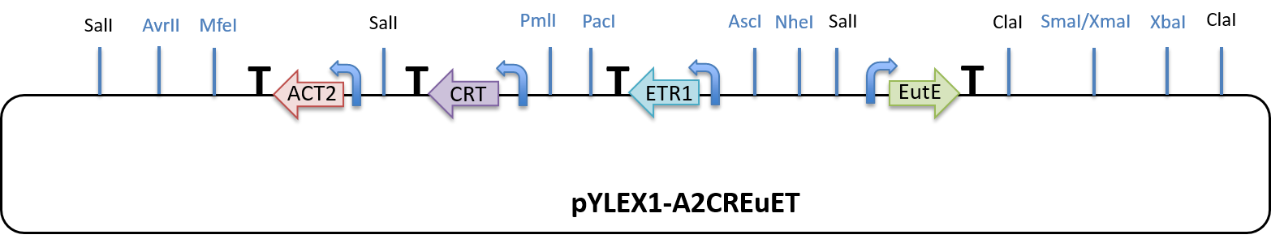


**Figure S5.** Map of the plasmid pYLEX1-A2CREuET


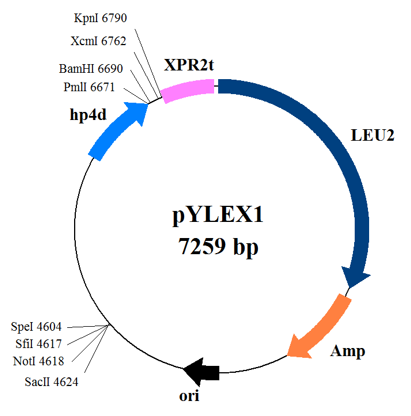

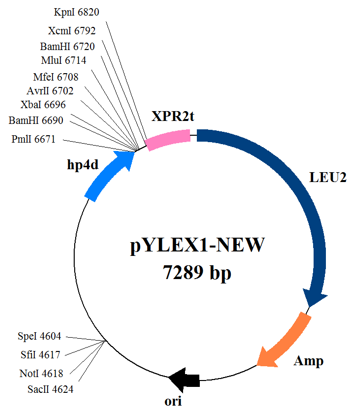


**Figure S6.** Restriction map of pYLEX1-NEW. Plasmid pYLEX1-NEW with an improved version of MCS was constructed by adding Xba I, Avr II, Mfe I and Mlu I sites into the BamH I site of pYLEX1. Plasmid pYLEX1-NEW contains two BamH I sites.
